# A review on the synthesis of bio-based surfactants using green chemistry principles

**DOI:** 10.1007/s40199-022-00450-y

**Published:** 2022-10-03

**Authors:** Shea Stubbs, Sakib Yousaf, Iftikhar Khan

**Affiliations:** School of Pharmacy and Biomolecular Sciences, Liverpool John Moores University, Liverpool, L3 3AF UK

**Keywords:** Alkyl polyglucosides, Bio-based surfactants, Green chemistry, Sucrose esters, Surfactants

## Abstract

**Objectives:**

With increasing awareness of the potential adverse impact of conventional surfactants on the environment and human health, there is mounting interest in the development of bio-based surfactants (which are deemed to be safer, more affordable, are in abundance, are biodegradable, biocompatible and possess scalability, mildness and performance in formulation) in personal care products.

**Method:**

A comprehensive literature review around alkyl polyglucosides (APGs) and sucrose esters (SEs) as bio-based surfactants, through the lens of the 12 green chemistry principles was conducted. An overview of the use of bio-based surfactants in personal care products was also provided.

**Results:**

Bio-based surfactants are derived primarily from natural sources (i.e. both the head and tail molecular group). One of the more common types of bio-based surfactants are those with carbohydrate head groups, where alkyl polyglucosides (APGs) and sucrose esters (SEs) lead this sub-category. As global regulations and user mandate for sustainability and safety increase, evidence to further support these bio-based surfactants as alternatives to their petrochemical counterparts is advantageous. Use of the green chemistry framework is a suitable way to do this. While many of the discussed principles are enforced industrially, others have only yet been applied at a laboratory scale or are not apparent in literature.

**Conclusion:**

Many of the principles of green chemistry are currently used in the synthesis of APGs and SEs. These and other bio-based surfactants should, therefore, be considered suitable and sustainable alternatives to conventional surfactants. To further encourage the use of these novel surfactants, industry must make an effort to implement and improve the use of the remaining principles at a commercial level.

**Graphical abstract:**

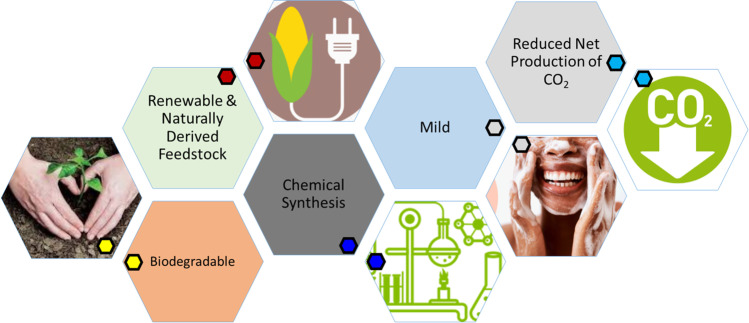

**Supplementary Information:**

The online version contains supplementary material available at 10.1007/s40199-022-00450-y.

## Introduction

The personal care industry includes the research, development and commercialization of consumer products used for cosmetic and personal hygiene purposes. This market is comprised of sub-categories such as oral care, deodorants and antiperspirants, hair and skin care, and color cosmetics. This diverse portfolio calls for a wide range of raw materials capable of fulfilling efficacy and marketing claims. Amongst these, surfactants are the most commonly used components [1]. The word surfactant is a combination of the words “Surface Active Agent” and is at times used interchangeably with the word amphiphile, due to its two-part structure. The polar head region of a surfactant is hydrophilic, whilst the tail of the molecule is hydrophobic and commonly comprised of a branched, aromatic or linear hydrocarbon chain. This unique structure enables it to lower the surface or interfacial tension between two liquids, a liquid and gas or liquid and solid [2]. When describing the boundary between a liquid and gas (usually air), the term surface tension is used; interfacial tension is used when describing that of two immiscible substances [3]. The ability to reduce the surface or interfacial tension is the most important property of the surfactant when used in personal care products, as it stabilizes the final preparation, produces a homogenous mixture, and allows it to effectively execute its primary function [4].

Conventional surfactants are comprised of oleochemicals (tail group), and petrochemicals (head group) [5, 6]. Once connected by a chemical bond, the two parts work together to display amphiphilic properties. Whilst oleochemicals can be derived from renewable crops such as coconut, palm and palm kernel oil; petrochemicals are sourced from finite fossil fuel reserves, and are used in larger quantities [5, 6]. Surfactants of this nature may be biodegradable, but can persist for extended periods of time, thus contributing to water and air pollution [7]. The availability and fluctuating costs of feedstock, coupled with an increased user interest in ingredients that are mild, sustainable and environmentally friendly, have led to the pursuance of bio-based surfactants by industry [5, 8]. Some surfactant manufacturers and researchers use this term to describe surfactants that are synthesized from microorganisms, whilst others say bio-based surfactants are those that have both their head and tail regions sourced from renewable feedstock. In this review, the latter definition is used. The use of oleochemicals for the tail group of the surfactant has occurred for decades. Therefore, the novel feature of bio-based surfactants is the synthesis of a renewable head group that can be effectively bonded to the tail region. Such molecules have been found to have great cleansing and foaming abilities, be extremely mild in formulation, biodegradable and benign [9–12].

The feedstock for the head group of bio-based surfactants is usually a peptide, amino acid or carbohydrate [13]. At the commercial level, carbohydrates are a leading source for the head group due to their price, availability and provision of flexible properties [5, 10]. This review will therefore cover alkyl polyglucosides (APGs) and sucrose esters (SEs)—two existing personal care bio-based surfactants with sugar head groups. Both of these surfactants have been successfully commercialized, are readily biodegradable, biocompatible, and offer the same, or comparable performance to petrochemical surfactants in personal care formulations [14, 15]. Presently, APGs and SEs are marketed as natural and/or green ingredients. Whilst the aforementioned characteristics support the claim that APGs and SEs are natural/green ingredients, as regulation and sustainability concerns of the user evolve, additional evidence to support the green identity of these surfactants will prove invaluable. Reviewing the production of these surfactants in the scope of green chemistry is one way to do this.

**Fig. 1:**
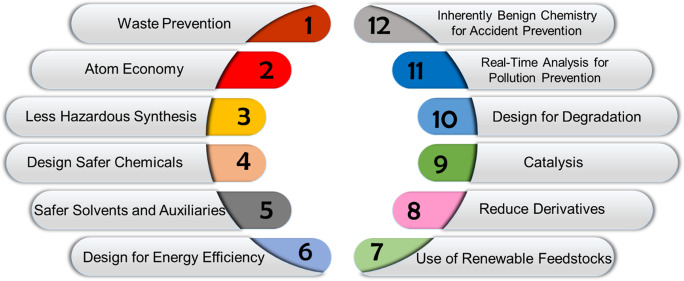
The twelve principles of Green Chemistry including; waste prevention, atom economy, less hazardous synthesis, design safer chemicals, safer solvents and auxiliaries, design for energy efficiency, use of renewable feedstocks, reduce derivatives, catalysis, design for degradation, real-time analysis for pollution prevention, and inherently benign chemistry for accident prevention

Shortly after the introduction of the Pollution Prevention Act in 1990, the Environmental Protection Agency (EPA) created the term ‘Green Chemistry’, describing it as the “design of chemical products and processes that reduce or eliminate the use and generation of hazardous substances” [16]. Using this definition, the twelve principles of green chemistry (Fig. 1) were devised as a guide for industry chemists and engineers [17]. Green Chemistry is driven by the concept of design. The principles challenge the industry to shift their habits of design with the goal of safety and sustainability in mind. This framework is meant to be applied to the entire lifecycle of the finished good – from raw material selection to chemical transformation, and ultimately the effect of the final product on human life and the environment [18]. The EPA calls for the fulfillment of these objectives whilst also producing profitable and functional products [19].

The driving forces behind the development of bio-based surfactants coincides with several of the green chemistry principles, particularly those related to feedstock. However, to be sustainable and lessen the adverse impact of industry on the environment, process and chemical design must consider factors beyond raw material source. This work therefore aims to examine the traditional synthesis routes of APGs and SEs through the lens of all of the elements of green chemistry. In this regard, a description of the feedstock production, reagents and processes are reviewed in the context of the relevant principles. An assessment of these factors and their impact on the final product on human life and the environment is conducted, and ultimately, support is given for the extent of greenness claimed, and opportunities for improved sustainability are highlighted.

## Conventional surfactants and surfactants with carbohydrate hydrophiles

Surfactants have many applications in cosmetic and pharmaceutical products. Their possession of both hydrophilic and lipophilic components enables their surface activity. When two immiscible substances (e.g. oil and water) are mixed, two distinct layers are formed as result of surface tension generated by their interface. When a surfactant is added to the container, it orients itself at the water–oil interface with the hydrophilic head group in the aqueous phase, and the hydrophobic tail group in the lipophilic phase [20]. The intermolecular forces between the surfactant and the water are much weaker than that between the water molecules (bulk phase), so the tension at the surface is lowered, making the substances miscible [21]. The higher the concentration of the surfactant in the mixture, the lower the surface tension. Upon addition of surfactants at higher concentrations, micelles may form [22]. The concentration and temperature above which this occurs are known as the critical micelle concentration (CMC), and krafft temperature respectively [22]. It is also at the CMC that a minimum surface tension is achieved [23]. The shape of the aggregate formed upon the addition of a surfactant is mainly dependent on the surfactant’s Hydrophilic-Lipophilic Balance (HLB); which is the ratio of the hydrophilic portion to the hydrophobic portion. This value can be calculated using the molecular structure of the surfactant. Hydrophilic surfactants have higher HLB values (> 10), and Lipophilic surfactants have lower HLB values (< 7), and are therefore suitable for oil-in-water and water-in-oil emulsions, respectively.

Molecules that possess these properties, and are derived in whole or significantly from biological products or renewable domestic agricultural or forestry materials, are known as bio-based surfactants [24]. This definition is interpreted in literature and in industry in numerous ways. This review considered all surfactants which possess a head and tail group sourced from renewable feedstock as bio-based. Although surfactants with head groups derived from petrochemicals remain the most acquired, demand for bio-based surfactants is likely to increase due to increasing sustainability demands from the user, their associated reduced net production of CO_2,_ and limits on fossil fuel sources [25].

**Fig. 2:**
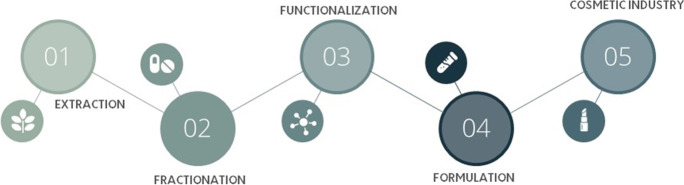
A schematic overview of the extraction of surfactant starting material from biomass

Bio-based surfactants with carbohydrate sourced head groups are one of the most important classes of surfactants [7, 26]. From both an economic and ethical standpoint, carbohydrates are ideal feedstock for chemicals. This is primarily because they have a natural hydrophilicity, are affordable, abundant and reproducible [25, 27, 28]. Carbohydrates are often represented by four groups: sugar, starch, cellulose and hemicellulose, and are sourced from a variety of plants (corn, beets, wheat etc.). To prepare for use in surfactant synthesis, biorefineries typically carry out a series of extraction, fractionation, and functionalization processes (bio-based surfactants present and future, Redon) (Fig. 2).

Surfactants with sugar head groups are the most popular, as they can be more stable when compared to those that are petrochemically sourced, and are capable of adequately lowering the surface tension at low concentrations [8, 14]. Biocompatibility and high biodegradability are also significant properties of surfactants sourced from sugar. With head groups sourced from glucose and sucrose, APGs and SEs are two of the main sugar containing surfactants produced at the industrial level in the personal care industry, and were therefore analyzed in this review [25].

## Alkyl polyglucosides (APGs)

Traditional APGs have a glucose head group derived from corn, and a fatty alcohol tail group that is derived mainly from palm kernel oil. Apart from their renewable feedstock, they are often preferred as surfactants due to their superior biodegradability, dermatological and ocular safety [29]. In the personal care industry, they act as cleansing agents, foam stabilizers, and rheology modifiers. In the past five years, they were primarily used in rinse off products (i.e. shampoo, body wash etc.) claiming to be “natural” [30]. The name APGs is given, as the product of the Fischer glycosidation mechanism contains alkyl mono-, di-, tri- and oligoglycosides as a combination of α- and β-anomers [31]. The APG is identified based on the length of the alkyl chain and the degree of polymerization.

**Fig. 3:**
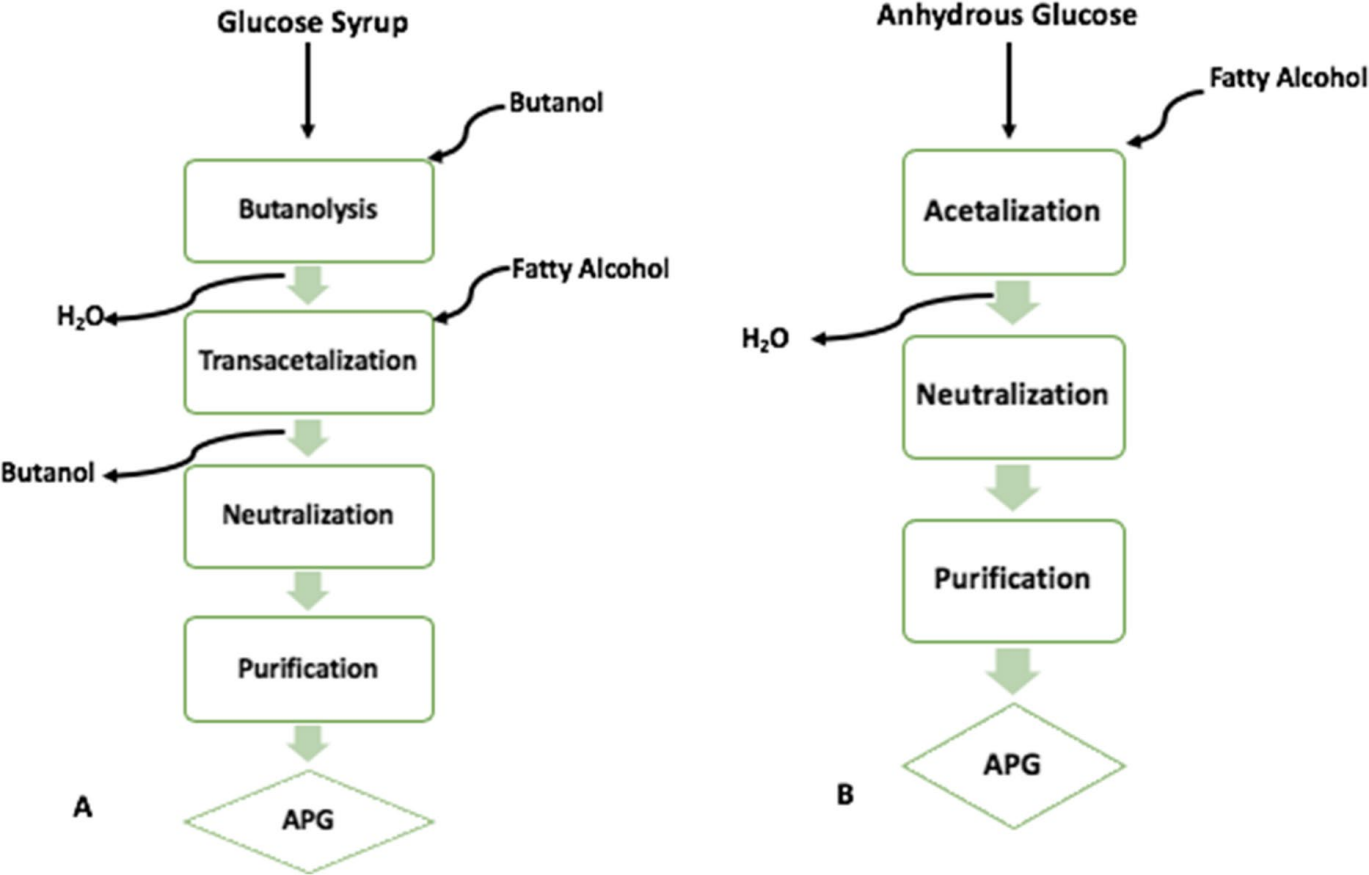
Synthesis route of alkyl polyglucosides (APGs) using various stages from (A) direct and, (B) indirect method

Industrially, the Fischer glycosidation reaction is conducted *via* a direct or indirect pathway between glucose and fatty alcohols (Fig. 3) [15]. During the early stages of development, the latter was very common as an affordable glucose syrup or starch can be used, whereas the direct acetalization requires anhydrous glucose, which can be expensive [10]. The first step in the indirect process is a butanolysis reaction. In the lab, this process begins by dissolving glucose in butanol in the presence of a weak acid. Para toluene sulphonic acid (PTSA) is often used. Water is produced at this step, so it is followed by the azeotropic removal at 105 ºC for 1 h [32]. The fatty alcohol is then added (in molar excess) slowly with additional PTSA while the temperature is increased in increments to 115–120 ºC. This step takes place under a vacuum of 300 mm Hg to remove the excess butanol [32]. Once the removal is complete, sodium hydroxide is added to neutralize the catalyst. The resulting mixture may contain excess fatty alcohol, which can be removed (to < 1%) via distillation at 80 ºC, under high vacuum or extraction with hexane and acetone [33]. The product is then dissolved in water to remove unreacted glucose and extracted with diethyl ether [32].

Although the indirect method offers cost benefits, there are manufacturers who prefer the direct method, owing to reduced need for equipment and efficiency in terms of time associated with the method. In this process the glucose is immediately reacted with the fatty alcohol via acetalization in the presence of an acid catalyst. As previously mentioned, anhydrous glucose is required; this is to minimize the side reactions that can occur in the presence of water [34]. The product is then neutralized to form the APG. In the direct method, a special distillation technology is implemented to remove excess fatty alcohol, followed by dissolution with water to remove glucose and bleaching for refinement [31, 35].

## Sucrose esters (SEs)

SEs have a sucrose head group derived from sugar cane, and a fatty acyl tail group sourced primarily from coconut oil. Traditional commercial routes form mono-, di-, tri- and higher esters. This trait gives a wider range of HLB values than other surfactants derived from molecules with multiple hydroxyl groups; making SEs suitable as emulsifiers in both oil-in-water and water-in-oil personal care emulsions [36, 37]. The CMC values of SEs are generally one to two magnitudes lower than that of other commercial surfactants. This is advantageous for cleansing products, as the lower the CMC value, the easier it is for the surfactant to form micelles, which increases overall formulation stability [38]. SEs also possess a high foam quality, and are mild, making them especially suitable for preparations such as baby shampoos [39]. In recent years however, SEs have been used mainly to formulate face/neck care (moisturizers and masks etc.) and body care products (balms, lotions etc.), with beauty enhancing properties (mattifying and pore reducing etc.) claiming to be naturally derived [40].

**Fig. 4:**
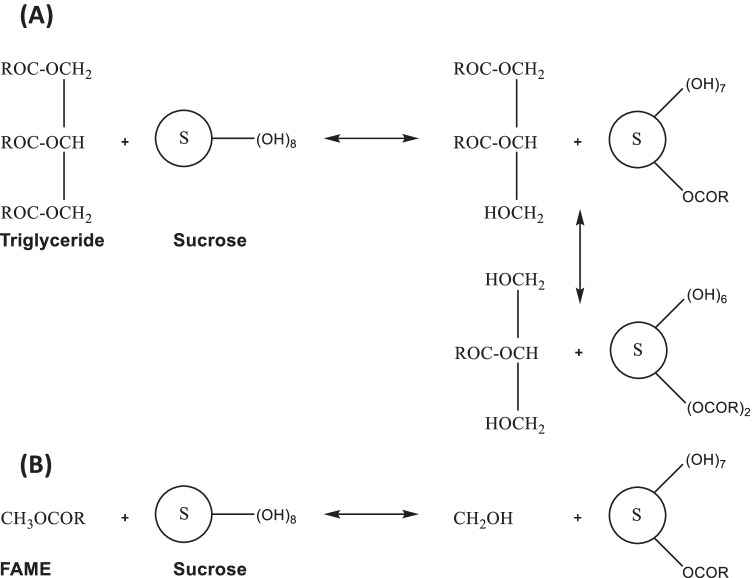
Graphical representation of;, (A) esterification of triglyceride with sucrose, yielding a mixture of mono- di- and higher esters and, (B) transesterification of fatty acid methyl ester (FAME) with sucrose, a most common synthesis route at the industrial level, yielding monoesters as the main product, and methanol is formed as a by-product

The manufacture of SEs can be challenging, as sucrose is temperature sensitive, and selectivity is challenging due to its eight hydroxyl groups [41, 42]. The most feasible way to synthesize SEs at an industrial level is by the esterification of triglycerides (Fig. 4A) or transesterification of fatty acid methyl ester (FAME) with sucrose in the presence of a basic catalyst (Fig. 4B) [31, 41]. The latter is generally preferred as it does not produce water and avoids the saponification of the triglyceride by the base catalyst [43]. This pathway will therefore be evaluated in this review.

The process starts with the preparation of a sucrose solution with Dimethyl Formamide (DMF) or Dimethyl Sulfoxide (DMSO). The sucrose-solvent mixture is then refluxed at 90–95 ºC. Once the solution is clear, the FAME and potassium carbonate catalyst are added [44, 45]. Reaction progress is determined with thin layer chromatography (TLC). The mixture of SE is then cooled to room temperature. Purification is achieved by neutralizing the catalyst using oxalic/lactic acid, brine washing, and evaporation [37, 44]. The final product typically contains 70% monoester and 30% di-, tri-, and higher esters [41].

## Green chemistry

Progress in terms of scientific developments has come at a cost of damage to the environment. Industrial chemical processes have led to more evident climate change e.g. holes in the ozone layer and a decline in forest health [17, 46]. During the twentieth century, these issues came to a head, forcing governments and agencies to address the need to balance economic and social growth, with the protection of the environment and human health. Through this need, disciplines such as green chemistry have emerged.

Green Chemistry promotes processes which conserve energy, prevent the formation of waste, and utilize catalysis routes. It also encourages the reduction or replacement of organic solvents with water or solventless processes. To accomplish these goals, industry has implemented techniques such as sourcing new raw materials from biomass, use of alternative reaction conditions, and non-toxic reagents [16, 18, 46]. It is important to note that green chemistry is not a separate science, but a sustainable approach to existing methods.

Sustainable development is defined by the World Commission on Environment and Development as development that meets the needs of the present, without compromising the needs of future generations [47]. Movement towards renewable feedstock, such as glucose and sucrose, is one aspect of this. The parameters of synthesis routes of bio-based surfactants, such as energy and water use, manufacturing processes and final product impact must also be addressed when attempting to achieve sustainability. The twelve principles of green chemistry act as guidelines for chemists and engineers to design chemicals with these factors in mind.

## Twelve principles of green chemistry

### Waste prevention

**Fig. 5:**
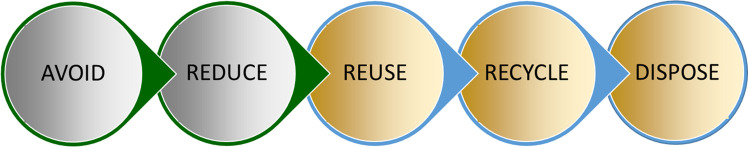
Pollution prevention hierarchy demonstrating disposal, recycling and reuse of waste are acceptable, but reduction and avoidance are preferred in the scope of green chemistry

The first principle of green chemistry is rooted in the philosophy that it is better to be proactive than reactive; it explains that technologies and procedures should be put in place to prevent or reduce waste through chemical and process design [17]. Where this is not possible, reuse, recycle and/or safe disposal of waste should be practiced (Fig. 5). The Environmental Factor (E-factor) metric is often used to determine the amount of waste produced by a process. It is calculated by dividing the mass of waste by the mass of desired product. The higher the E-factor, the greater the amount of waste produced [46]. In the chemical industry, higher E-factors are generally a result of use of organic solvents and stoichiometric reactions. Catalysis and solvent-free processes should therefore be explored to reduce waste, and thus reduce environmental impact [48]. Lower E-factors contribute to reduced manufacturing costs due to reduced disposal, better utilization of capacity, and lower energy use. There is therefore potential for the bio-based surfactant industry to offset increased raw material costs by implementing this green chemistry principle.

#### Alkyl polyglucosides (APGs)

The major outputs in the synthesis of APGs are water, glucose, fatty alcohol and butanol (indirect method only). Literature discusses the continuous removal of water and excess fatty alcohol for recycling and reuse [31, 49]. While this is more efficient than direct waste disposal, it is not waste prevention. These steps therefore do not comply with this principle of green chemistry. To maximize efficiency and prevent additional waste generation, vapor pressure and vapor–liquid equilibrium data for products should be collected. This will assist with determining the relative volatility, and thus, the best way to approach the separation [50].

A large quantity of glucose in the final product is a result of the reaction ending prematurely [35]. Ways to prevent this are not described, however, analytical methods should be put in place to verify completion of the reaction prior to neutralization of the acid catalyst. Additionally, chemists should consider redesigning the molar ratios of the starting material. Implementation of such procedures should minimize the amount of glucose waste produced. Similar to other waste products in the routes described, specific handling methods of the butanol by-product are not mentioned. However, where possible, this material should be recycled or appropriately treated and disposed.

#### Sucrose esters (SEs)

Prior to purification, the SEs may be accompanied by solvent, unreacted sucrose and fatty acid methyl ester (FAME) [37]. Methods involving a continuous basis approach have been developed to minimize the formation of these materials [51]. With this technique, unreacted ester fractions, sucrose and fatty acid ester are continuously recycled until reaction completion. While this reduction of waste is favorable, it is not considered prevention. To increase sustainability, literature suggests enzymatic synthesis. Candida antarctica type B lipase (CALB) is often the biocatalyst used industrially. This technique facilitates regioselectivity, which maximizes the amount of SE, and thus prevents waste formation [52].

Methanol is formed as a byproduct in traditional SE routes; this drives the equilibrium in favor of the ester, thus improving the yield of the final product [41, 43, 53]. Processes, such as distillation, are used to continuously remove the methanol. Although it does not end up in the final mixture, it is also not a part of the final product and cannot be reused, so it is considered waste. Molar ratios should be designed to minimize the formation of this byproduct, while maintaining maximum yield of SE.

### Atom economy

Historically, the efficiency of the reaction has been determined through calculation of product yield (weight of actual product divided by theoretical product weight). While this is helpful, it does not provide a full picture of what has occurred, as most chemical transformations are not as simple as “reagent A converted to product B”. It is therefore possible to calculate a high product yield and the process still be inefficient due to a formation of waste [19]. To account for this, green chemistry proposes the use of atom economy. This metric gives a ratio of the molecular weight of the desired product to the sum of the molecular weights of all substances produced. In doing so, a better indication of the efficiency of the process is given [48]. Both yield and atom economy should, therefore, be considered when designing the process.

#### Alkyl polyglucosides (APGs)

The butanol and water formed as by products in the indirect method reduce the atom economy of the reaction. Using the example of lauryl glucoside (C_18_H_36_O_6_) and assuming 100% yield, an atom economy of 79% is given. Whilst this is not extremely inefficient, 21% of waste can be costly at the industrial level. In the direct method, lauryl glucoside can be produced at an atom economy of 95%. This reaction is much more efficient, as only water is formed as a byproduct. These values do however decrease in the likely event that the yield of the reaction is less than 100%. For example, Wang et al. [54] synthesized lauryl glucoside at a yield of 96% with a 7:1 fatty alcohol to glucose ratio. In this instance, the atom economy would be 91%. It is therefore important to design a process that includes reagents and techniques that allow for the highest yield possible, with the least amount of waste (Supplementary Materials (S1 and S2)).

#### Sucrose esters (SEs)

The conventional pathway for the synthesis of SEs is a reversible reaction. The route that involves reacting FAME with sucrose is preferred at the industrial level, as methanol formation drives the equilibrium in favor of the SE, which increases the yield of the desired product [41]. Using the example of sucrose laurate, and assuming 100% yield, the atom economy of this route is 94%. The final product may, however, contain mono-, di- and triesters. If all three esters are not the desired product, this may lower the atom economy. To avoid this, sucrose is often modified before reacting with FAME, to allow for the formation of a single desirable product rather than multiple esters (Supplementary Materials (S3 and S4)).

### Less hazardous chemical synthesis

In chemical manufacturing, the starting material is converted into a number of intermediate compounds before being transformed to the final product. The substances generated, and the reagents used to generate them, should have little to no physical hazards such as explosivity and flammability [19].

#### Alkyl polyglucosides (APGs)

To improve the safety around APG synthesis, industries should reevaluate the use of the indirect pathway, as it requires the use of butanol to form the butyl glycoside intermediate. Butanol is extremely flammable, and may release vapors that are explosive indoors, outdoors and in sewers [55]. Using a solvent of this nature in large quantities undermines the safety of the manufacturing process. The use of the direct method, or other butanol-free pathways should be explored to apply this green chemistry principle. It should however be noted that attempting to increase the safety in this way may increase the manufacturing costs due to the anhydrous glucose requirement.

#### Sucrose esters (SEs)

During the synthesis of SEs, the methanol by-product must be continuously removed to accommodate efficient progression of the reaction. This is a flammable liquid, so special care must be taken during the removal steps of the process. Distillation is often used to accomplish this [41]. Additionally, the combination of relatively high reaction temperatures, with homogenous alkaline catalysts, and DMF/DMSO may lead to safety and environmental issues [9]. While this reaction is described as being controlled, these factors prevent complete compliance with this green chemistry principle.

### Design safer chemicals

When assessing the risk of a chemical, its intrinsic hazard and the exposure to that hazard are generally the two areas of consideration. The fourth principle of green chemistry addresses the intrinsic hazards by highlighting the structural-activity of a chemical and how it may be physiologically hazardous [19]. As a result, it urges manufacturers to pay special attention to the design of chemicals, ensuring that the final product is effective, but non-toxic. This principle is extremely important when it comes to the personal care industry, as the products are created to have relatively high levels of exposure to the user. Although rinsed off, shampoo, shower products and liquid soap are in contact with the scalp, arms, legs, face, and possibly eyes, on a regular basis. Surfactants must therefore be evaluated to determine their compatibility with these areas over time.

#### Alkyl polyglucosides (APGs)

When tested for acute toxicity after ingestion and contact with skin under the Organization for Economic Cooperation and Development (OECD) guidelines (No. 41 and 42), and Toxic Substances Control Act (TSCA) regulations, short (C_8/10_) and medium (C_12/14_) chain APGs (most common in cosmetics), were proven to be non-toxic [34]. Personal care products are often used daily, so it is important to take this evaluation further by examining the sub chronic toxicity, or the adverse effect caused by repeated, daily application. C_12/14_ APGs were therefore tested over a 90-day period in male and female Wistar rats under the OECD guideline No. 408. This test concluded that a daily dose of 1000 mg/kg over this period of time did not lead to any toxic effects; this dose is listed as the “no observed adverse effect level” (NOAEL) [35, 56]. APGs (C_12/14_) were also found to not be skin sensitizing when tests were conducted under the OECD guideline No. 406 – a result that is supported by human patch tests [34].

Assessment is not as straight forward when it comes to dermal and mucous membrane irritation. For example, C_8/10_ APGs in commercial concentrations were found to be non-irritating, while C_12/14_ APGs of the same concentration were (OECD test No.404). Thus, indicating an effect of alkyl chain length on dermal irritation. Under a 30% concentration however, these surfactants did not elicit a response. It was therefore concluded that concentration of APG must also be considered when assessing dermal irritation [34, 56]. The same was discovered during mucous membrane tests. In Draize procedures, a sample of 0.1 mL APG solution is applied to the eyes of at least 4 rabbits, and contact is permitted for 24 h. After recording responses over a 21-day period, C_8/10_ APGs were found to be non-irritating to the eyes, while C_12/14_ APGs were determined to be irritating [34, 56]. This outcome, and the others above indicate that APGs have an acceptable safety profile, but caution must be taken when creating formulations with APGs of certain alkyl chain lengths. Although APGs are non-toxic and are used at much lower concentrations than 30% in formulation, manufacturers must be aware of the structural impact on irritation, and reflect this awareness during synthesis, analytical and purification steps.

#### Sucrose esters (SEs)

A safety assessment was conducted by the Cosmetic Ingredient Review (CIR) Panel on 40 saccharide esters, 32 of which contained sucrose head groups. To do so, the toxicity, genotoxicity, carcinogenicity, irritation and sensitization data were evaluated. Where gaps were found, read-across based on structural similarities was conducted. In animal and human tests, no dermal irritation was found for most esters, and mild irritation found in sporadic cases. The panel therefore deemed the potential of dermal irritation to be low [36]. This conclusion is supported in other published sources by Ayala-Bravo et al. [57] and Pyo et al. [37]. They also found through both *in-vitro* and an *in-vivo* tests that this group of chemicals are non-sensitizing. Similar to the APGs however, an exception is noted for medium chain esters. Literature reports that SEs with a linear C_12_ chain are better as penetration agents when compared to longer chain esters [57, 58]. This may be attributed to associated intermediate HLB values which allow the SE to penetrate the lipid bilayer. These traits, along with results of medium cell viability and release of interleukins in their experimental methods, led to relative toxicity and skin sensitizing potential conclusion for sodium laurate. This SE is however, still used safely. The CIR panel simply cautions formulators when developing cosmetic preparations. The remaining SEs were categorized as non-toxic – a conclusion that is supported by other published toxicity studies [36, 59, 60].

Most of the SEs evaluated by the CIR panel were found to be non-irritating to the eyes. Here again, sucrose laurate (C_12_) was the exception. At a concentration of 10%, this surfactant showed slight irritation in rabbits. It is however reported, to be used in much lower concentrations in cosmetic preparations [36]. These results indicate that the final design of SEs is acceptable in the scope of green chemistry. Knowledge of the effect of alkyl chain length on the interaction of this group of surfactants with the stratum corneum should be further evaluated, and possible modifications explored to ensure continued safe use in cosmetics.

### Safer solvents and auxiliaries

In chemical manufacturing, solvents and auxiliary substances, such as carrier fluids and separation agents, are often introduced to form the desired product. The mass of these materials tend to exceed that of the synthesized compound by as much as 100-fold [19]. Handling such volumes exposes manufacturing staff to increased risks, while contributing to waste. This principle therefore calls for industry to reinvent their process, in an effort to reduce, replace or remove non-participatory chemicals. Ideal green alternatives are nontoxic, from renewable sources, affordable and readily available [61].

#### Alkyl polyglucosides (APGs)

In the indirect method of APG synthesis, diethyl ether may be used in the final step for extraction. While effective at recovering the final product, this chemical is extremely volatile due to its low flash point. The Environmental, Health and Safety (EHS) group at Pfizer Global Research and Development, designed a solvent selector tool in effort to assess the suitability of solvents based on worker, process and environmental safety. In doing so, they advise against the use of diethyl ether and suggest replacement with 2- methyl tetrahydrofuran or tert-butyl methyl ether. While these solvents are both flammable and have other drawbacks, they are much more usable than diethyl ether on the industrial scale [62].

Alternatively, manufacturers can consider using the direct method, where water is the only solvent used to remove unreacted glucose. The distillation technology used in this process allows for effective removal of the water, so that the final product is produced without the need of an extraction step [63]. Again, the glucose used in this method is less affordable than that in the indirect method, but the opportunity to avoid additional solvents may be advantageous in the long run from an economic and green chemistry standpoint.

#### Sucrose esters (SEs)

Although an effective solvent for the synthesis of SEs, DMF was found to be extremely hazardous due to its combustible nature, and ability to react violently with oxidizing agents, alkali metals, carbon tetrachloride, and chlorinated hydrocarbons. DMF is also easily absorbed through the skin, and is known to be toxic to the liver due to long-term exposure [64]. Risks are therefore high for employees that would have to handle this solvent in large volumes daily. Many manufacturers have therefore applied this principle of green chemistry by replacing DMF with DMSO, as it is non-toxic and less expensive [41, 44].

DMSO does however, have its handling drawbacks. Therefore, to avoid using either of the conventional solvents, a microemulsion based process, known as the Nebraska-Snell reaction, was developed. Years later, Yamagishi et al. [65] made changes to this process to make it more feasible at the industrial scale. Here, propylene glycol, a “generally recognized as safe” solvent, is used, and aqueous sucrose and fatty acid soap is reacted with a fatty acid ester in the presence of an alkali catalyst. The reaction facilitates the formation of a microemulsion, which prevents sucrose hydrolysis and other side reactions [37]. The elevated temperatures at which the reaction takes place (110–175 ºC) may lead to the caramelization of sucrose, thus reducing the yield. Although movement away from toxic solvents is an improvement, business (yield) and green chemistry (additional energy required for the higher temperatures) compromises are made. Kidani et al. [66] suggested a solution to this through microwave heating at 90 ºC. With this method, higher yields of monoester with no discoloration or odor (two common quality issues) are formed in around half the time as that of conventional heating [66, 67].

While the microwave method is effective, it still requires a solvent. Today, many manufacturers avoid such routes due to the exhaustive separation methods to receive a pure final product, and the amount of solvent required (60%) for a successful transformation. This has therefore led to the development of solvent-less processes to synthesize SEs [43]. With these techniques, a slurry of sucrose is reacted with FAME in the presence of potassium carbonate and an emulsifier (either fatty acid carboxylate or SE) at 130 ºC. While they may be safer to conduct and economical, the final product of solvent-free methods is often only suitable for use in specific applications, such as detergent formulations [41]. Research to broaden the performance of SEs made from solvent-less processes should be prioritized, in an effort to increase sustainability in this regard.

### Design for energy efficiency

Most synthetic chemical reactions require heat for the transformation to take place at a reasonable rate. The amount of energy needed to accommodate this at the industrial scale may have a negative impact on the environment, and can significantly increase manufacturing costs [19]. This green chemistry principle, therefore, calls for the synthesis of chemicals at an ambient temperature and pressure.

#### Alkyl polyglucosides (APGs)

Unfortunately, neither of the traditional synthesis routes for APGs are capable of these conditions. In the indirect method, the glucose syrup requires depolymerization in the first step, which requires temperatures > 140 ºC. This elevated temperature can lead to higher pressures, which can overwork the equipment leading to an increase in production costs [35]. The need to make this method more economical led to the development of the direct method. This was accomplished through the optimization of reaction temperature, pressure, reaction times and fatty alcohol to glucose ratios [31]. The direct synthesis of APG, therefore occurs at lower temperatures (100–120 ºC), however these are still much higher than the desired ambient temperature.

To avoid high temperatures and pressures over extended periods of time, microwave technology has been used as an alternative energy source. This method allows for efficient energy transfer, rapid heating, and monitoring of the energy output. As a result, the process is more controlled and atom economy is increased through the reduction of side reactions. Zhou et al. [68] applied this technology to the conventional synthesis routes of APGs and have reported accelerated reactions times (minutes vs. hours) and α-glycoside product selectivity. In their analysis, they also mention that microwave equipment can be added to continuous flow systems, with little or no need for optimization, allowing for feasible transfer of energy source at the industrial level [69].

#### Sucrose esters (SEs)

SEs are typically synthesized at temperatures of 90–140 ºC and under atmospheric and/or vacuum pressures [44, 45, 70–72]. Although vacuum pressures are lower than atmospheric pressure, the pump required to create these conditions requires a lot of energy. Temperatures in both pathways are limited to ranges under 160 ºC, as at this temperature, the sucrose begins to caramelize [43, 73]. Therefore, most researchers aim to stay within the 90–95 ºC range. Although this is not considered elevated (> 100 ºC), this is far above the ambient temperature, and requires more energy than deemed ideal by the principles of green chemistry. At room temperature however, this reaction is extremely slow, and longer reaction times may lead to more overall energy used in the process [73].

To address the temperatures required for the transformation, Cruces et al. [74] have created a simple transesterification procedure involving DMSO, vinyl ethers and a disodium hydrogen phosphate catalyst, which can take place at 40 ºC and at atmospheric pressure. This method produced yields higher than 85%, with a higher percentage of monoesters than SEs commercially available [74]. Additional work is required however, to make this and other safer, greener routes (enzymatic synthesis, use of ionic liquids etc.) cost effective at the industrial scale.

### Use of renewable feedstocks

The seventh principle of green chemistry calls for the chemical industry to use renewable raw materials wherever technically and economically feasible, as there are a lot of environmental and political issues surrounding the production and use of petroleum [46].

#### Alkyl polyglucosides (APGs)

As bio-based surfactants, APGs, from a top-level view, abide by this principle. The industry, however, must be cautious not to take an oversimplified approach to this matter. While there are many drawbacks to relying solely on petroleum as a raw material, it would be careless to say that a chemical is sustainable simply because it is derived from renewable crops. The glucose used for the hydrophilic portion of APGs can be extracted from wheat and potatoes, but is typically derived from corn [10]. Although corn is a renewable feedstock, the technologies used to grow this crop and obtain glucose can be counter to two major objectives of green chemistry: the protection of the environment and waste prevention.

Agricultural practices to grow corn typically include the use of chemical fertilizers, pesticides, and energy intensive harvesting and transportation; all of which can be harmful in various ways [63]. For example, nitrogen fertilizers are often used, but are both soil and plant soluble, making them susceptible to run-off. Eventually, these fertilizers make it to bodies of water where they contribute to eutrophication. This overgrowth of algae can disrupt wildlife and lead to the production of toxins that may be harmful to humans. The overuse of water is also a concern in the production of corn for glucose. Pesticides are unable to target kill, potentially killing useful organisms in soil, causing a deterioration in quality. As a result, water retention decreases and a lot more water is required to produce the same amount of crop overtime. Waste in this form is not consistent with green chemistry principles.

These factors have encouraged the surfactant industry to begin exploration of new sources for the APG head group. For example, the use of pentose as an alternative to glucose has been proposed. Pentose can be sourced via the transglycosylation of lignocellulosic carbohydrates (e.g. wheat). This feedstock utilizes 37–41% less fertilizers and 36–57% less nonrenewable energy than the conventional glucose-based process. Using this route yields a mixture of APGs and alkyl polypentosides (APP). The presence of APPs makes the final surfactant more lipophilic than traditional APGs, which contributes to a lower CMC. Generally, the lower the CMC, the more efficient the surfactant and the more advantageous the economics on the industrial scale. Additionally, APPs have been found to contribute to lower toxicity [75].

The fatty alcohol for the surfactant tail is obtained by the transesterification of triglycerides derived mainly from palm kernel and coconut (C_12-14_ fatty alcohols), or, palm and rapeseed (C_16-18_ fatty alcohol) feedstock [63]. Palm kernel is however, the most commonly used, and will therefore be discussed in this review. The overall environmental impact of APGs is significantly affected by fatty alcohol production. In fact, in life cycle assessment (LCA) studies, where effects of a chemical from cradle to grave are examined, fatty alcohols have been found to contribute more to global warming (kg CO_2_) than the growth of corn crop and extraction of glucose [75, 76]. In another LCA study, a comparison of palm kernel derived fatty alcohol and petrochemical derived fatty alcohol are presented [77]. The latter performed better in 12 out of the 18 environmental impact categories evaluated (Table 1). It should however be noted that these results are primarily due to palm mill operational processes, such as the amount of land used, use of fertilizers, pesticides, soil health and growth (and thus, CO_2_ absorption) [77]. If these are adjusted, palm kernel oil derived fatty alcohol remains a suitable renewable alternative for surfactant feedstock. In an attempt to make this adjustment, surfactant manufacturers often certify their palm and palm kernel by-products with non-profits such as the Roundtable on Sustainable Palm Oil (RSPO). This organization provides a certification that ensures credibility of sustainability claims by members of the supply chain. To increase the sustainability of the synthesis of the fatty alcohol, research on bioconversion methods, such as metabolic engineering, as alternatives to chemical synthesis have been conducted [78]. Such methods use less energy, do not require toxic catalysts, and have less greenhouse gas emission than the chemical processes. They are however, much slower, have lower yields and require purification steps.

**Table 1:** Results from an environmental impact evaluation of palm kernel oil derived fatty alcohol vs petrochemical derived fatty alcohol are given, where the later performed better in twelve of the eighteen impact categories

Impact category	Average PKO fatty alcohol performance	Average petrochemical fatty alcohol performance
Agricultural land occupation		✓
Climate Change		✓
Fossil depletion	✓	
Freshwater ecotoxicity		✓
Freshwater eutrophication		✓
Human toxicity	✓	
Ionizing radiation	✓	
Marine ecotoxicity		✓
Marine eutrophication		✓
Metal depletion	✓	
Natural land transformation		✓
Ozone depletion	✓	
Particulate matter formation		✓
Photochemical oxidant formation		✓
Terrestrial acidification		✓
Terrestrial ecotoxicity		✓
Urban land occupation		✓
Water depletion	✓	

#### Sucrose esters (SEs)

Both the head and tail group in SEs are sourced from renewable feedstock, but, similar to the APGs, it is worth evaluating the cultivation methods of these crops before deeming the feedstock sustainable. Sucrose is readily available at extremely high purity and low cost, as it is one of the highest volume organic compounds in the world [41]. Eighty percent of sucrose is derived from sugar cane, with the remainder from sugar beet [79]. This discussion will therefore primarily focus on the cultivation of sugarcane for SE hydrophiles.

During the harvesting of sugarcane, the leaves and cane tops are disregarded, as they are low in sucrose content but contain a high amount of starch and reducing sugars, which can lower the sucrose yield [80]. The removal process is done by hand or fire. The canes are then cut by hand (most common) or using mechanical harvesters. In order to utilize the manual harvesting technique, the sugarcane must first be burnt. Harvesting cane that has not been burnt is less economical and can be hazardous to plantation staff [81]. This pre-harvest burning, contributes to air pollution by adding particulate matter, carbon monoxide, and hydrocarbons to the atmosphere; all of which negatively impact the respiratory systems of employees and members of nearby communities [81]. The lime kilns, evaporator stations and vacuum boilers are also potential sources of volatile organic compounds (VOC) [80]. With the evaporators however, the gases released are recycled and used to power other evaporators in the series, thus reducing the amount of pollution and energy consumption. Other methods such as cyclone systems are also used to control the exhaust from machinery [80].

The use of chemical fertilizers and pesticides may also have a negative impact on the environment. Only a fraction of these substances is taken up by the crop. The remainder run-off into the environment, generally ending up in bodies of water, increasing aquatic pollution. Literature therefore recommends cutting cane in the field, as cane trash (tips, leaves etc.) is known to be rich in nutrients that can reduce the need for chemical fertilizers. The use of renewable fertilizer and a more precise placement of chemical fertilizer where needed, can assist with incorporation of green chemistry practices to this process [81, 82].

### Reduce derivatives

Organic molecules often have more than one mechanistic pathway. To ensure that a specific pathway is selected, chemists and engineers often introduce protection groups to the process. In doing so, the starting material is modified so that undesired reactions are blocked, and the desired mechanism can occur. While work has been done to make this an efficient part of organic synthesis, it generally creates more waste, as the protection groups must be removed and disposed of after serving their purpose [46]. Adding protection groups also adds extra steps to the process, which requires longer reaction times, and thus, more energy is used.

#### Alkyl polyglucosides (APGs)

When using glucose as a reagent, multiple pathways are available, and must therefore be synthesized via selective glycosylation. This requires activation methods, protective groups, or selective catalysis by enzymes [35]. As discussed previously, on the industrial level, acid catalyst activation is most common, thus avoiding the inefficiencies of derivatization, and complying with this principle of green chemistry.

#### Sucrose esters (SEs)

The sucrose molecule contains nine chiral centers and eight hydroxyl groups, which include three primary hydroxyls (carbons 6,1’ and 6’) and five secondary hydroxyls (carbons 2,3,4,3’ and 4’). Theoretically, esterification with an equal molar amount of reagent should therefore form eight sucrose monoesters [83]. Some of the hydroxyls (at carbons 6 and 6’) are more reactive than others, but the product formed is still contaminated with other monoesters. Therefore, to make the reaction most efficient, research has been done on modification methods to ensure that a single product is formed [41]. A number of studies report on the introduction of derivatives to accomplish this via both chemical and enzymatic synthesis routes [41, 84–86]. While relatively high yields of monoester have been isolated with these pathways, the scale up of these methods are not always economical, so the esterification method followed by purification remains the commercial route; in the scope of green chemistry (less steps and reduced reaction time), this pathway is preferred [37, 83].

### Catalysis

The use of stoichiometric reagents is often associated with the production of waste in industrial synthesis. For this reason, the use of catalytic reagents is therefore deemed more efficient by the principles of green chemistry. Additionally, catalysis increases reaction rates, selectivity and reduces the number of mechanism steps. Catalysts may also be recycled, thus increasing the affordability of the process [87].

#### Alkyl polyglucosides (APGs)

The conventional synthesis of APGs always requires an acid catalyst. PTSA is typically the catalyst of choice for both methods, as it is low in cost, and soluble in water, alcohol and organic solvents. Other organic acids have been utilized, but PTSA remains a favorable green heterogeneous catalyst, as it is efficient and can be reused [88, 89]. It is also non-oxidizing to butanol, not corrosive on reactors, and considered a weak acid, which makes the neutralization step simple when producing APGs [90]. Enzyme catalysts have also been applied to the synthesis of this surfactant in an effort to increase selectivity, reduce energy and possibly reduce the volume of solvents used. As it stands however, the cost and ability to recycle, make PTSA the more attractive green option at the industrial scale.

#### Sucrose esters (SEs)

Catalysis is also the most practical way to synthesize SEs in the described routes [73]. In the past, alkaline metal hydroxides have been used as catalysts, but as previously mentioned, water is produced, which may cause soap formation, and thus a lower yield. Yields are improved by using K_2_CO_3_ as an alternative, as it allows for the formation of bicarbonate instead of water [44, 91]. It is also favored for synthesis reactions due to its mild nature, ability to produce good yields and accommodate short reaction times [92]. While effective, selectivity remains an issue with K_2_CO_3._ Enzymes have been proposed as an efficient solution, as they have the ability to direct the substitution for the acyl group on the sucrose molecule in mild, environmentally friendly conditions [93]. Lipases and proteases are often the enzymes of choice [71]. As with the APGs however, additional research is required to implement this method at the industrial level due to high costs of enzymes, low yields and extensive reaction times, when compared to the base-catalyzed process [37, 71, 91]. The same can be said for other emerging techniques such as the use of ionic liquid-based catalysts.

### Design for degradation

The environmental compatibility of chemicals is often determined by their level of biodegradability. The tenth principle of green chemistry advises chemists and engineers to design products so that they do not persist in the environment but break down into benign products.

#### Alkyl polyglucosides (APGs)

APGs are used mostly in rinse-off personal care products such as shampoos and body washes. Therefore, after use, the products enter domestic wastewater, which eventually infiltrates larger bodies of water. This review will therefore focus on APGs interaction with the aquatic environment, instead of the terrestrial environment. The OECD scheme produces data for aerobic (in the presence of oxygen) and anaerobic biodegradation of a chemical. Both parameters are equally as important, as personal care surfactants often end up in areas with little to no oxygen such as septic tanks or polluted rivers [94]. Steber et al. [95] found that C_12/14_ and C_8/10_ APGs have a high degree of ultimate biodegradability, after conducting the Closed Bottle Test, Modified OECD Screening Test and Dissolved Organic Carbon (DOC) Die-Away Test over a 28-day period. The OECD’s pass level for ready biodegradability is 60% for the Closed Bottle Test and 70% for the Modified OECD Screening and DOC Die-Away Tests. The APGs tested far surpassed these limits with results of 88% (Closed Bottle) and 90% (Modified Screening & DOC) [35, 95].

Although the same results were collected for C_12/14_ and C_8/10_ APGs, studies have shown that biodegradability varies by initial concentration, alkyl chain length and number of glucose units [96]. For example, in screening tests conducted by Jurado et al. [97] results indicated that the lower the initial concentration of APG, the higher the biodegradation (> 90%). It was however also determined by this same group, that APGs with the longest alkyl chain and highest number of glucose units had the lowest biodegradability levels, regardless of initial concentration [97]. Branched APGs were also found to be more persistent in anaerobic conditions than liner APGs. They were however, found to have very low aquatic toxicity, so this result is of little concern [98]. Ultimately, scientific studies show that contamination of bodies of water and negative impact on aquatic life by APGs is unlikely as their high biodegradation levels suggest that they will biodegrade in municipal and domestic digesters prior to arrival into the environment [15]. This generally occurs in a multistep process, which begins with hydrolysis – yielding an alcohol and polysaccharides (Table 2). The alcohol is then oxidized to carbon dioxide and water via β or α oxidation, while the polysaccharides are hydrolyzed to glucose. Ultimately, the glucose is broken down via a metabolism reaction [63]. Therefore, the APGs that happen to come into direct contact with river sediment and soils are still expected to have little effect, as this proposed pathway indicates that both linear and branched APGs of varying concentrations biodegrade wholly to CO_2_ and water—making them green surfactants, sufficiently satisfying the tenth principle of green chemistry [63, 97, 99].

**Table 2:** Summary of synthesis, degradation pathway, biocompatibility and personal care application of Alkyl Polyglucosides (APGs) and Sucrose Esters (SEs)

Category	Bio-based Surfactant		Key Studies
	Alkyl Polyglucosides (APGs)	Sucrose Esters (SEs)	
*Synthesis*	Indirect or Direct Fischer Glycosidation	Esterification of Triglycerides or Transesterification of FAME	***APGs:*** [15, 32, 34] ***SEs:*** [41, 42, 44]
*Degradation* *Pathway*	Multi-step: 1.Hydrolysis to form alcohol and polysaccharides 2a. Oxidation of alcohol 2b. Hydrolysis of polysaccharides	Oxidation of alkyl chain or Hydrolysis of the ester bond	***APGs:*** [63] ***SEs:*** [100]
*Biocompatibility*	Acceptable safety profile (non-toxic, non-irritating and non-skin sensitizing) – but caution must be taken when formulating with APGs of certain alkyl chain lengths	Acceptable safety profile with exception of the medium chain esters	***APGs:*** [34, 35, 56] ***SEs:*** [36, 37]
*Personal Care Applications*	Cleansing Agent Emulsifiers Foaming Enhancers and Stabilizers Rheology Modifiers	Cleansing Agent Emulsifiers	***APGs:*** [34, 63, 101, 102] ***SEs:*** [37, 71]

#### Sucrose esters (SEs)

One of the reasons SEs are attractive as surfactants is because of their ready, rapid biodegradability into benign end products. While the literature on degradation is not as extensive for this surfactant as it is for APGs, multiple, reputable sources have reported and/or found, *via* International Standard Organization (ISO) methods, that SEs are biodegradable in both anerobic and aerobic conditions [37, 71, 74, 100, 103]. This generally occurs in one of two pathways; the first involves the oxidation of the alkyl chain, and the other is a hydrolysis reaction of the ester bond, followed by independent degradation of the hydrolysis byproducts [100]. The level and the period of degradation by either pathway was found to vary based on the structure of the SE (Table 2).

To increase the water solubility of the SE in formulations, anionic derivatives are often synthesized by adding an α-sulfonyl group. Baker et al. [100] found however, that the presence of this side group significantly reduced the biodegradability of the surfactant. In their evaluation, sucrose laurate rapidly degraded in one day, while its sulfonated analog, sucrose sulfonyl laurate, was only 84% biodegradable after a 25-day period. They conclude that the presence of the sulfonyl group in the α position stabilizes the ester bond, making the hydrolysis pathway more difficult. To remain compliant with this principle, chemists should explore alternative methods to increase solubility.

The same study looked at the effect of head group size, number of alkyl chains attached to the head group, and alkyl chain length, and concluded that changes in these features do not have a significant effect on biodegradability. Other studies do however report a slight effect on the length of the alkyl chain, but the results are conflicting. Either way, Baker et al. [100] noted that any effect would likely be insignificant in the environment, as biodegradation is more probable than in a laboratory setting. To ere on the side of caution, manufacturers of SEs with longer alkyl chain lengths should investigate further.

### Real-time analysis for pollution prevention

The objective of this principle is to encourage the development of analytical methods to allow for immediate, in-process analysis of the batch. In doing so, unfavorable features are detected and corrected before the end of the reaction, thus minimizing the final amount of pollution from the process.

#### Alkyl polyglucosides (APGs) and sucrose esters (SEs)

Process Analytical Technology (PAT) is an example of a system used in many industries that is capable of designing, analyzing and controlling the manufacturing through measurements at specific times in throughout the process [104]. Whilst this type of technology is not explicitly mentioned in literature for the indirect synthesis of APGs at the industrial level, Estrine et al. [63] mentioned the use of a process information system for the direct method by Cognis—one of the leading manufacturers of APGs. Lack of detail concerning this principle is also apparent for SE production. To ensure alignment with this principle however, chemists and engineers should research and develop technology to monitor methanol formation, the presence of contaminant, and optimize/alter molar ratios.

There is undoubtedly a gap in the literature for the application of this practice to the traditional production of both APGs and SEs, which may or may not be an indication of a gap of this principle in industry. Incorporating this type of analysis is straightforward from an engineering standpoint, but from a chemistry perspective may require a lot of redevelopment. For many manufacturers, this may not be worth the investment, due to the fact that the processes have evolved, and are now relatively controlled and low on waste formation [19].

### Inherently benign chemistry for accident prevention

**Fig. 6:**
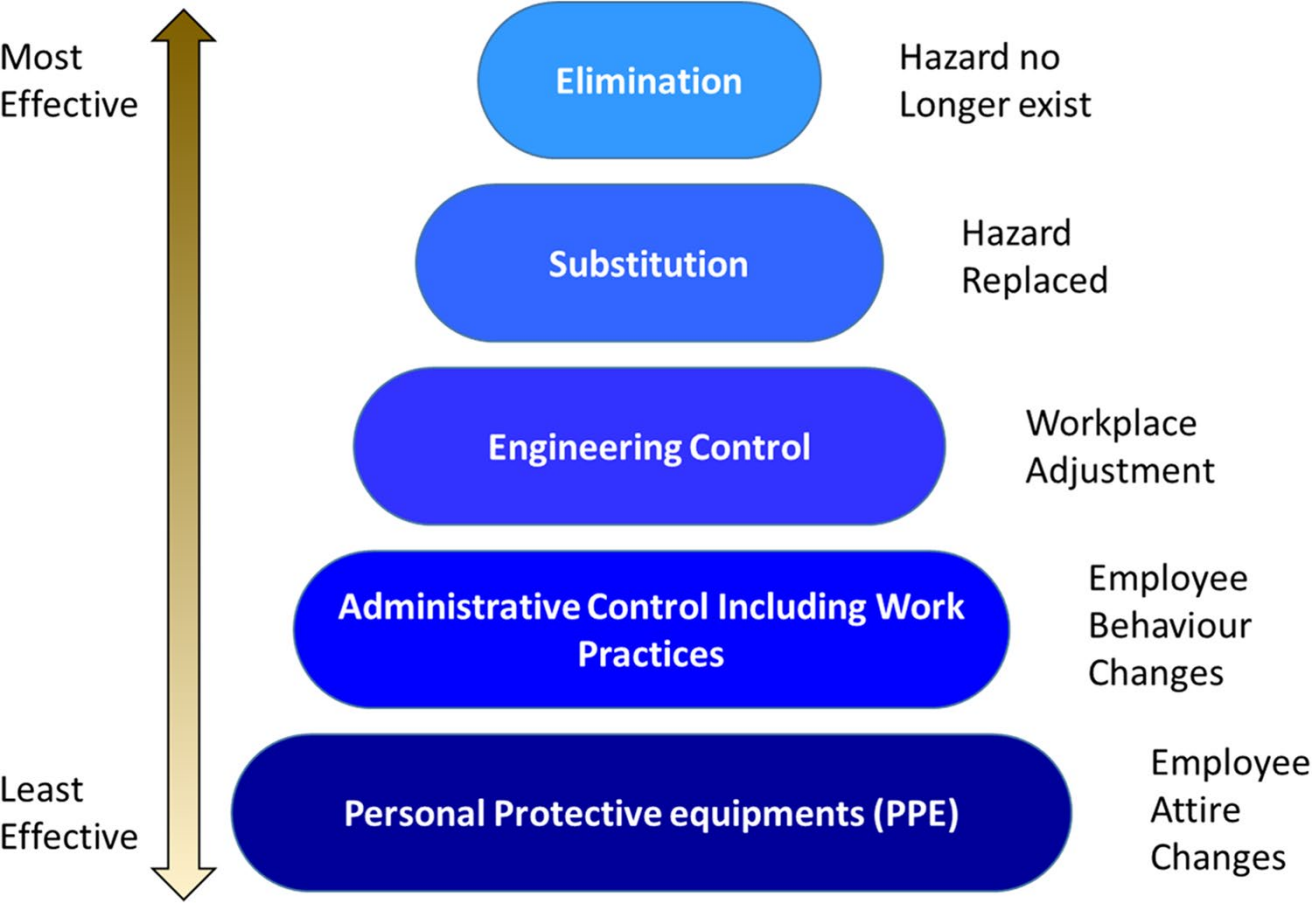
Hazard control hierarchy, where elimination is the goal, but substitution, engineering control, safe work practices and personal protective equipment are also accepted

The final principle of green chemistry is concerned with the safety of the manufacturing staff. There have been countless scenarios of explosions, injuries and other tragedies at chemical facilities due to the improper handling and/or nature of dangerous materials. This principle therefore urges manufacturers to consider a transition from flammable, reactive materials to protect personnel and the surrounding communities. Elimination or replacement of the hazard in this way is ideal, but not always possible. In these instances, other acceptable, though less effective, methods of protection such as, readjusting of the facility, safety checks and use of personal protection equipment should be utilized (Fig. 6).

#### Alkyl polyglucosides (APGs)

The process for the production of APGs is inherently safe but the use of materials such as butanol and sodium hydroxide should be evaluated. Although not used in molar excess, a sufficient amount of butanol is required to dissolve glucose in the first step of the indirect synthesis of APGs. As a result, plant workers must handle tons of this very flammable chemical, that may produce poisonous gases if a fire occurs. Additionally, butanol will react with oxidizing agents, alkali metals, and alkaline earth metals to produce an explosive hydrogen gas [105]. These risks should prompt APG manufacturers to consider transitioning to the direct method, which does not call for the use of this chemical.

Sodium Hydroxide is not flammable, but must still be highlighted, as it is very reactive. This material reacts with strong acids and water to form heat that can cause severe burns. It can also react with metals such as zinc and lead to form an explosive hydrogen gas. Literature does not indicate if safer routes have been established to protect staff. These changes can increase production costs and may not be able to occur immediately for various supply chain and operational reasons. Companies must therefore ensure that workers are aware of and compliant with the appropriate Occupational Safety and Health Administration (OSHA) and the National Institute for Occupational Safety and Health (NIOSH) exposure and threshold limits at all times. Workers must also be encouraged to revise the supplier’s safety data sheet for specific handling information before each use.

#### Sucrose esters (SEs)

As previously mentioned, DMSO is preferred as a solvent over DMF. Although less toxic, in both liquid and vapor form, DMSO is combustible. Once the reaction is complete, the mixture generally contains 70% DMSO prior to separation [37]. A multi-stage solvent removal process to get the desired product is therefore required. A lot of risk is associated with having manufacturing staff conduct a separation series involving a combustible material. Where possible, methods discussed above which involve safer or no solvent should be utilized. If this is not possible, procedures and/or technology should be implemented to reduce the amount of isolation steps and minimize the amount of handling time by personnel.

## Personal care applications

### Alkyl polyglucosides (APGs)

#### Surfactant (Cleansing agent)

APGs have many properties that make them suitable ingredients in the personal care industry. Due to their use in products such as shampoos, body and facial washes; cleansing is certainly the most important property. Rinse-off personal care cleansing products have been historically prepared with anionic surfactants such as sodium lauryl sulfate and sodium laureth sulfate (SLES) [106]. Although extremely effective, many users have found these ingredients harsh and irritating. As a result, decyl, lauryl and coco glucoside have become increasingly popular due to their ability to provide a deep yet gentle clean to the hair and skin over a broad range of pH levels [34, 56, 101, 107]. This mildness is a result of the compatibility of these surfactants with the epidermis. When a formulation containing SLES and APG was evaluated under the parameters of the Modified Duhring Chamber Test, the erythema score was found to decrease with an increasing percentage of APG. In fact, development of irritation was determined to be reduced by 20–30% when 25% of SLES was replaced by decyl glucoside [34]. This supports the use of this group of molecules not only as great primary surfactants, but also as excellent co-surfactants in cleansing formulations where mildness is desired.

#### Emulsifiers

Emulsions are the most common vehicle for cosmetic preparations. Formulating them, however, can be challenging as they are thermodynamically unstable. Emulsifiers stabilize the system, but are often the most irritating excipient in the formulation [108]. When surfactants are used as emulsifiers, they have the potential to interact with the skin lipids, which damages the barrier. There has therefore been lots of research in effort to identify emulsifiers with good safety profiles that can effectively moisturize the skin and provide stability. Longer chain APGs were found to fulfill these criteria. After cleansing, emulsifying is said to be the next most important function of this group of surfactants [102]. When compared to ethoxylated surfactants, APGs provide stability to water/oil emulsions that is independent of temperature and low interfacial tension [63, 101]. Additionally, there are studies that recognize APGs ability to protect the skin from known irritants (acidic acids, SLS etc.) and quicken skin barrier repair [109].

#### Foaming enhancers and stabilizers

Foaming is another extremely important characteristic of personal care products, as users often associate superior cleansing with the amount/quality of foam produced. APGs, particularly C_12_/C_14_ cuts, display exceptional foaming abilities when compared to fatty alcohol ethoxylates in similar formulations [63, 101]. The foam volume increases with decreasing length of the hydrocarbon chain. Due to their foam enhancing abilities, APGs are often used with other surfactants, such as acyl glutamates to increase foam volume. When this is done, the final formulation produces a foam equally as abundant as high foaming alkyl sulfates. With the help of APGs, formulators are therefore able to produce mild cleansing products with comparable foaming abilities as products made with harsher surfactants [102].

Anionic surfactants are extremely high foaming, particularly in the presence of sebum and hard water. APGs are often used as co-surfactants with anionic surfactants to stabilize this foam formation [83]. In doing so, 20% less surfactant is used in the formula to produce the same foaming power when used without APGs. For example, when SLES is used individually, it produces a coarse, dry, unstable foam. APGs, such as lauryl glucoside, typically form a fine, wet, creamy foam. Therefore, when used together in formulation, a stable foam is produced [102].

#### Rheology modifiers

In recent years, personal care raw material manufacturers have developed surfactant concentrate blends. These products are optimized blends of surfactants designed as complete preparations for a specific purpose. With these blends, cosmetic finished goods manufacturers can achieve a final product by simply diluting with water, adjusting the viscosity, and adding desired additives. These concentrates are particularly attractive to smaller companies or brands as they reduce inventory costs, cycle time, and are typically cold processed; all of which produce a cost-effective formulation [34].

While surfactant blends are extremely effective, they tend to have high viscosities, and therefore issues of transfer from raw material supplier packaging to the customer’s equipment are likely to arise. This may result in product loss and an increase in production time – challenges that defeat the purpose of concentrate use. APGs have been found to address these issues. Upon addition to surfactant concentrates containing up to 60% actives, APGs modify the rheology, making them pumpable and ready for dilution [35]. For example, most ether sulfates are in the gel phase at 30–45% active, making use in large quantities difficult [102]. When a blend of an ether sulfate is made in a 2:1 ratio with C_12/14_ APGs, the APG prevents the development of the viscous hexagonal phase of the ether sulfate, allowing for pumping above 15 °C and standard dilution methods [35]. In addition to ether sulfates, APGs are usually present in surfactant concentrates with betaines and other non-ionic surfactants. These blends are typically used in shampoo and shower product formulations that aim to be milder on the eyes and skin [34].

### Sucrose esters (SEs)

#### Cleansing agent

SEs, particularly those with 12 or more carbons, are known to be effective cleansing agents due to their surface tensions of 25–40 mN/m and their broad range of CMC values, which are generally one to two magnitudes lower than that of other commercial surfactants [37, 41]. This is advantageous for cleansing products, as the lower the CMC value, the easier it is for the surfactant to form micelles, which increases overall formulation stability [38]. Additionally, SEs have high foam quality, and are mild, making them especially suitable for preparations such as baby shampoos [39].

#### Emulsifiers

When sucrose is used as the feedstock for the hydrophilic component of the surfactant, mono-, di-, tri- and higher esters are produced. This trait gives a wider range of HLB values than other surfactants derived from molecules with multiple hydroxyl groups [36, 37, 110]. SEs with high HLB values (> 11) have a high composition of monoesters and are suitable to stabilize oil-in-water emulsions. Those with lower HLB values (5–7) are rich in di- and triesters and may be used to form water-in-oil emulsions. The fatty acid chain length of the SE also has an effect on the emulsifying properties, due to its effect on HLB. The shorter the chain, the higher the HLB value. These higher HLB SEs are unique, as they are able to form emulsions with small droplets, which increases the overall stability. They are also great for preparations where a creamy skin-feel is desired [71]. The versatility in HLB provided by the chemical design of SEs allows for emulsification in a variety of preparations such as sunscreen, creamy lotions, and gels [37].

### Other personal care bio-based surfactants

#### Sorbitan esters

The personal care surfactant industry has manufactured sorbitan esters for decades with volumes of approximately 20,000 tones/year [111, 112]. This non-ionic surfactant is typically synthesized via direct esterification of sorbitol with fatty acids and an alkaline catalyst. This mechanism often yields a mixture of mono-, di- or trisorbitan esters with a HLB range of 1–8, making them suitable water-in-oil emulsifiers [10, 111]. They can also be used as co-emulsifiers with high HLB surfactants in oil-in-water preparations. Sorbitan stearate and sorbitan isosterate are recorded as most used in personal care, functioning mainly in creams and lotions, often for users with sensitive skin [113].

#### Polyglycerol esters

Glycerol is one of the main byproducts in oleochemistry, however, is not capable of acting as the hydrophile in a bio-based surfactant. It is therefore polymerized in the presence of a base catalyst to form a polyglycerol. This head group then undergoes esterification to synthesize a polyglycerol ester surfactant [10]. These molecules are favored in many industries due to their multifunctionality, mild nature, biodegradability and biocompatibility. Polyglycerol esters vary in composition depending on the method of manufacture. Only recently have manufactures manage to achieve high purity products, making this non-ionic surfactant suitable for food and personal care preparations [10, 114]. These ingredients function mainly as emulsifiers, but may also be used as dispersants, solubilizers, viscosity modifiers, emollients and conditioners in cosmetics [114, 115].

#### Fatty acid glucamides

When the reductive amination of glucose occurs with methyl amine, N-methyl glucamine is formed. Subsequently, this intermediate is reacted with a fatty acid methyl ester to form fatty acid glucamides. Although not wholly bio-based like the beforementioned surfactants, this sugar derived molecule is extremely mild, compatible with other surfactants and has a low irritancy profile [10]. Alkyl glucamides have historically been used in home care (i.e. detergents), but these characteristics have prompted their commercialization as personal care surfactants in recent years [111]. One major disadvantage that formulators must be cognizant of however, is their affinity to calcium ions. As a result, a sequestering agent must always be used to avoid precipitation of this surfactant once in formula.

## Conclusion

This review confirms the applicability of renewable feedstock, biodegradability, catalysis, atom economy and safe chemical design green chemistry principles to the traditional production of APGs and SEs. In doing so, support is given for the use of bio-based surfactants as greener alternatives to conventional surfactants in industry. The remaining green chemistry principles have yet to be scaled-up, are not adhered to, or have been excluded from the discussion of current practices. It is, however, possible that the principles of green chemistry are utilized in their entirety commercially and have been excluded from literature. To implement or to improve the use of the principles in the commercialization of APGs and SEs, and encourage the use of bio-based surfactants, industry must commit to a greater focus on the development of sustainable and innovative systems, business practices and technology.

## Supplementary Information

Below is the link to the electronic supplementary material.

Supplementary file1 (DOCX 17 KB)
