# Supplementary material for: A review on the synthesis of bio-based surfactants using green chemistry principles
Source: Daru. 2022 Oct 3;30(2):407–26. doi: 10.1007/s40199-022-00450-y (PMC9715898; doi:10.1007/s40199-022-00450-y)
Supplement: Supplementary file 1 — Supplementary file1 (DOCX 17 KB) [file 40199_2022_450_MOESM1_ESM.docx]

**Atom economy**

**S1**. Molecular weights of desired product and total products formed in indirect synthesis of lauryl glucoside (C_18_H_36_O_6_), and the equation below demonstrate the atom economy calculation of lauryl glucoside. Assuming 100% yield, the atom economy of this reaction is 79%.

| Desired Products | | All Products | |
| --- | --- | --- | --- |
| Formula | Weight (g/mol) | Formula | Weight (g/mol) |
| C_18_H_36_O_6_ (lauryl glucoside) | 348.48 | C_18_H_36_O_6_ | 348.48 |
|  |  | C_4_H_10_O | 74.12 |
|  |  | H_2_O | 18.02 |
| Total | **348.48** | **Total** | **440.62** |

$$Atom Economy \left( \% \right)= \frac{MW of desired product}{sum of MWs of all products} x 100$$

$Atom Economy (\%) =\frac{348.48}{440.63} x 100\boldsymbol{=79\%}$ **Yield**

**S2**. Molecular weights of desired product and total products formed in direct synthesis of lauryl glucoside (C_18_H_36_O_6_), and the equation below demonstrate the atom economy calculation of lauryl glucoside. Assuming 100% yield, the atom economy of this reaction is 95%.

| Desired Products | | All Products | |
| --- | --- | --- | --- |
| Formula | Weight (g/mol) | Formula | Weight (g/mol) |
| C_18_H_36_O_6_ | 348.48 | C_18_H_36_O_6_ | 348.48 |
|  |  | H_2_O | 18.02 |
| Total | **348.48** | **Total** | **366.50** |

$$Atom Economy= \frac{348.48}{366.50} x 100=\boldsymbol{95\%}\mathbf{Yield}$$

**S3**. Molecular weights of desired product and total products formed in direct synthesis of lauryl glucoside (C_18_H_36_O_6_) at 96% yield, and the equation below demonstrate the atom economy calculation of this reaction is 91%.

| Desired Products | | All Products | |
| --- | --- | --- | --- |
| Formula | Weight (g/mol) | Formula | Weight (g/mol) |
| C_18_H_36_O_6_ | (0.96) 348.48 | C_18_H_36_O_6_ | 334.54 |
|  |  | H_2_O | 31.96 |
| Total | **348.48** | **Total** | **366.50** |

$$Atom Economy= \frac{334.54}{366.50} x 100=\boldsymbol{91}\% \mathbf{Yield}$$

**S4**. Molecular weights of desired product and total products formed in direct synthesis of sucrose laurate (C_24_H_44_O_12_), and the equation below demonstrate the atom economy calculation of lauryl glucoside. Assuming 100% yield, the atom economy of this reaction is 94%.

| Desired Products | | All Products | |
| --- | --- | --- | --- |
| Formula | Weight (g/mol) | Formula | Weight (g/mol) |
| C_24_H_44_O_12_ | 524.60 | C_24_H_44_O_12_ | 524.60 |
|  |  | CH_3_OH | 32.04 |
| Total | **3524.60** | **Total** | **556.64** |

$$Atom Economy= \frac{524.6}{556.64} x 100=\boldsymbol{94\%}\mathbf{Yield}$$
